# Assessment of Isocitrate Dehydrogenase 1 Genotype and Cell Proliferation in Gliomas Using Multiple Diffusion Magnetic Resonance Imaging

**DOI:** 10.3389/fnins.2021.783361

**Published:** 2021-11-22

**Authors:** Yan Xie, Shihui Li, Nanxi Shen, Tongjia Gan, Shun Zhang, Weiyin Vivian Liu, Wenzhen Zhu

**Affiliations:** ^1^Department of Radiology, Tongji Hospital, Tongji Medical College, Huazhong University of Science and Technology, Wuhan, China; ^2^Magnetic Resonance Research, General Electric Healthcare, Beijing, China

**Keywords:** diffusion magnetic resonance imaging, glioma, isocitrate dehydrogenase, cell proliferation, multi-parameter magnetic resonance image

## Abstract

**Objectives:** To compare the efficacy of parameters from multiple diffusion magnetic resonance imaging (dMRI) for prediction of isocitrate dehydrogenase 1 (IDH1) genotype and assessment of cell proliferation in gliomas.

**Methods:** Ninety-one patients with glioma underwent diffusion weighted imaging (DWI), multi-*b*-value DWI, and diffusion kurtosis imaging (DKI)/neurite orientation dispersion and density imaging (NODDI) on 3.0T MRI. Each parameter was compared between IDH1-mutant and IDH1 wild-type groups by Mann–Whitney *U* test in lower-grade gliomas (LrGGs) and glioblastomas (GBMs), respectively. Further, performance of each parameter was compared for glioma grading under the same IDH1 genotype. Spearman correlation coefficient between Ki-67 labeling index (LI) and each parameter was calculated.

**Results:** The diagnostic performance was better achieved with apparent diffusion coefficient (ADC), slow ADC (D), fast ADC (D^∗^), perfusion fraction (f), distributed diffusion coefficient (DDC), heterogeneity index (α), mean diffusivity (MD), mean kurtosis (MK), and intracellular volume fraction (ICVF) for distinguishing IDH1 genotypes in LrGGs, with statistically insignificant AUC values from 0.750 to 0.817. In GBMs, no difference between the two groups was found. For IDH1-mutant group, all parameters, except for fractional anisotropy (FA) and D^∗^, significantly discriminated LrGGs from GBMs (*P* < 0.05). However, for IDH1 wild-type group, only ADC statistically discriminated the two (*P* = 0.048). In addition, MK has maximal correlation coefficient (*r* = 0.567, *P* < 0.001) with Ki-67 LI.

**Conclusion:** dMRI-derived parameters are promising biomarkers for predicting IDH1 genotype in LrGGs, and MK has shown great potential in assessing glioma cell proliferation.

## Introduction

In 2016, the World Health Organization classified levels of Central Nervous System tumors based on molecular features, specifically the isocitrate dehydrogenase 1 (IDH1) genotype (Karsy et al., 2017). IDH1 is a key enzyme involved in cellular metabolism, epigenetic regulation, redox, and DNA repair, existing in the cytoplasm and peroxisomes (Fu et al., 2010). Many studies have indicated a better prognosis of IDH1-mutant gliomas than IDH1 wild-type gliomas (Turkalp et al., 2014; Shen et al., 2020). Ye et al. (2013) found that IDH1 mutation causes a decrease in hypoxia-inducible factor −1α, which in turn leads to the inhibition of HIF-1α-mediated biological functions such as pro-angiogenesis, angiogenesis, migration, and motility of endothelial cells. Therefore, accurate identification of glioma IDH1 genotype facilitates the formulation of treatment plans and assessment of patient prognosis.

A nuclear protein, Ki-67, represents the proliferative activity of tumors and is closely associated with tumor differentiation and infiltration (Habberstad et al., 2011). The higher Ki-67 labeling index (LI) indicates faster tumor growth and poorer tissue differentiation. However, the cost of gene screening is high and not easy to broadly implement (Jiang et al., 2018). To obtain tumor pathology information and Ki-67 LI via surgery or pathological biopsy is not applicable for all gliomas, such as those in the brainstem and basal ganglia regions. Thus, an imaging approach to obtain anatomical details and tissue characteristics is essential for clinic diagnosis.

Diffusion magnetic resonance imaging (dMRI) is a non-invasive method to reflect microstructure information of diffusion of water molecules. The conventional mono-exponential diffusion-weighted imaging (DWI) reflects water motion paths *in vivo*. Diffusion tensor imaging (DTI) indicates the integrity of the white matter fiber tracts in the brain under the assumption of water random movements in Gaussian distribution (Maier et al., 2010). Since the complex cellular microenvironment in real organisms is limited by organelles, cell membranes, and extracellular gaps (Van Cauter et al., 2012; Jiang et al., 2015), water moves in non-Gaussian distribution and can be revealed by diffusion kurtosis imaging (DKI) (Wu and Cheung, 2010). DKI cannot reflect the intrinsic biophysical mechanisms such as the altered membrane permeability of axons, while neurite orientation dispersion and density imaging (NODDI) reflects a closer approximation to the real diffusion pattern of water molecules in the tissue microenvironment via characterizing the three main tissue cavities in the microstructural environment, namely, restricted diffusion of water within the neurite, hindered diffusion of water outside the neurite, and free diffusion of water in the cerebrospinal fluid (Zhang et al., 2012). In addition, the bi-exponential intravoxel incoherent motion (IVIM) imaging is able to separate the slow diffusive motion in response to intra- and extracellular water molecules as intra-tissue diffusion from the fast diffusive motion in response to intravascular water molecules as vascular perfusion (Shen et al., 2016), while the stretch-exponential DWI is able to describe the tissue heterogeneity and the continuous distribution of water molecules in the microstructure (Bennett et al., 2003).

Diffusion magnetic resonance imaging analyzed with different diffusion models reflects the complexity of tumor microstructure. Till now, few studies have been performed to compare the efficacy of these diffusion models in terms of IDH1 genotype. The purpose of this study was to assess IDH1 genotype and cell proliferation using mono-exponential, bi-exponential, stretch-exponential DWI, DTI, DKI, and NODDI models.

## Materials and Methods

### Patient Population

This study was approved by local ethics committee and written informed consent was obtained from all subjects. Patients were included in the study if they met the following inclusion criteria: (a) pathologically confirmed primary gliomas; (b) preoperative DWI, multi-*b*-value DWI, and DKI/NODDI acquisition were performed; (c) IDH1 genotype measured by genetic screening or immunohistochemistry was available. The following were exclusion criteria: (a) purely cystic gliomas; (b) lack of routine MRI images. From July 2017 to December 2020, a total of 94 patients meeting the above inclusion criteria were enrolled in the study. Two cases with purely cystic glioma and one lack of routine images were excluded. In total, 91 patients were recruited into this study.

### Image Data Acquisition

All MR images were performed on the a 3T MR system (Discovery MR750, GE Medical Systems, Milwaukee, WI, United States) with a 32-channel head coil. Routine axial sequences include T1 fluid-attenuated inversion recovery (FLAIR), T2 fast spin echo (FSE), T2 FLAIR, contrast-enhanced T1 weighted spin-echo.

Three diffusion imaging data were obtained using spin-echo echo-planar imaging sequences before the injection of contrast agents. The parameters of DWI were as follows: TR/TE = 3,000/70 ms, NEX = 1, matrix = 160 × 160, slice thickness = 5 mm, slice spacing = 1.5 mm, FOV = 240 mm × 240 mm, *b* = 0 and 1,000 s/mm^2^, and acquisition time was 42 s. Multi-*b*-value DWI was performed with 20 *b*-values (*b* = 0, 20, 50, 80, 100, 150, 200, 400, 600, 800, 1,000, 1,200, 1,500, 2,000, 2,400, 2,800, 3,200, 3,600, 4,000, and 4,500 s/mm^2^), for 0–1,000 s/mm^2^, NEX = 1, for 1,200–2,800 s/mm^2^, NEX = 2, and for 3,200–4,500 s/mm^2^, NEX = 4, TR/TE = 3,200/90.6 ms, matrix = 160 × 160, slice thickness = 5 mm, spacing = 1.5 mm, FOV = 240 mm × 240 mm, acquisition time was 5 min 52 s. DKI/NODDI was performed with 3 *b*-values (*b* = 0, 1,250, and 2,500 s/mm^2^) and 25 uniformly distributed directions for each nonzero *b*-value, TR/TE = 6,500/85, NEX = 1, matrix = 128 × 128, slice thickness = 3 mm, spacing = 0 mm, FOV = 240 mm × 240 mm, acquisition time was 5 min 45 s.

### Image Processing and Regions of Interest Analysis

Mono-, bi-, and stretch-exponential DWI, IVIM, DTI, DKI parametric maps were processed using GE Advantage workstation (version 4.5). After brain extraction of images by MRIcron (Version 12-12-2012), the NODDI parametric maps was processed using a MATLAB toolbox^1^.

The apparent diffusion coefficient (ADC) map was obtained by the following mono-exponential model:


S⁢(b)/S⁢(0)=e⁢x⁢p⁢(-b⋅A⁢D⁢C)

Where *S*(*b*) and *S*(0) represent the signal intensity for *b* = 0 and a non-zero *b*-value.

In bi-exponential intravoxel incoherent motion (IVIM) model, three maps of slow ADC (D), fast ADC (D^∗^), perfusion fraction (*f*) were processed:


$$S\,\left(b\right)/S\,\left(0\right)=\left[f\cdot e\,x\,p\,\left(-b\cdot {\text{D}}^{*}\right)\right]+\left[\left(1-f\right)\cdot e\,x\,p\,\left(-b\cdot \text{D}\right)\right]$$

The maps of distributed diffusion coefficient (DDC) and heterogeneity index (α) were derived using a stretched-exponential model:


$$S\,\left(b\right)/S\,\left(0\right)=e\,x\,p\,\left[-\left(b\cdot {\text{DDC}}^{\alpha }\right)\right]$$

The DDC, which represents mean diffusion rate within voxels, can be considered as a composite of individual ADC values. The α varying between 0 and 1 reflects the degree of tissue heterogeneity.

The DTI quantitative maps including fractional anisotropy (FA) and mean diffusivity (MD) were calculated in a Gaussian-distributed model:


$$S\,\left(b\right)/S\,\left(0\right)=e\,x\,p\,\left(-b\cdot D_{a\,p\,p}\right)$$

The value of *b* is 1,250 mm^2^/s in this study, and *D*_*app*_ is the ADC.

In addition, mean kurtosis (MK) map, a DKI parameter, was obtained in non-Gaussian-distributed model:


$$S\,\left(b\right)/S\,\left(0\right)=e\,x\,p\,\left(-b\cdot D_{a\,p\,p}+1/6\,b^{2}\cdot D_{a\,p\,p}^{2}\cdot k_{a\,p\,p}\right)$$

*K*_*app*_ is the diffusion kurtosis.

The NODDI model was processed using a MATLAB toolbox (see text footnote 1). NODDI with two shells (*b* = 1,250 and 2,500 mm^2^/s) could obtain orientation dispersion index (ODI) and intracellular volume fraction (ICVF) maps. The full normalized signal S in NODDI can be written as:


$$S=\left(1-V_{i\,s\,o}\right)\left(V_{i\,c}S_{i\,c}+\left(1-{\text{V}}_{i\,c}\right){\text{S}}_{e\,c}\right)+V_{i\,s\,o}{\text{S}}_{i\,s\,o})$$

Where V_ic_ and S_ic_ is the non-Gaussian volume fraction and intracellular diffusion signal, V_iso_ and S_iso_ is the normalized volume fraction and signal for the isotropic Gaussian diffusion compartment. And S_*ec*_ is the extracellular normalized signal.

All parametric maps and routine MR images were analyzed using ImageJ software (version 1.52a, NIH, United States). Before drawing the regions of interest (ROIs), the size (256 × 256), number of slices (Raab et al., 2010), as well as canvas size (240 mm × 240 mm) of routine sequences images and all parameter maps were adjusted to ensure they had the same image resolution, number of slices, and FOV. Two neuroradiologists (both with 5 years of experience in neuroradiology) who were blinded to clinical data manually placed three to six ROIs (range, 29–98 pixels) in consensus in the solid parts of tumor parenchyma and avoided hemorrhage, calcification, edema, necrosis, and cystic lesions. The solid part of tumor parenchyma was defined as the area of enhancement on contrast-enhanced T1-weighted images. If there was no enhancement on contrast-enhanced T1-weighted images, the solid part was defined as the area of abnormal signal on T2 FLAIR and T2 FSE. Then, the ROI was copied to each parameter map to obtain the measurements for each parameter. Similar to the previous studies (Figini et al., 2018; Wang et al., 2019), the minimum ADC, D, f, DDC, a, MD, and maximum D^∗^, MK, ODI, and ICVF from all ROIs for each patient were recorded.

### Statistical Analysis

Chi-square tests or R × C columnar tables were used to test categorical variables. Mann–Whitney *U* test was used to test continuous variables. Receiver operating characteristic (ROC) curves were performed to evaluate the diagnostic efficacy of each parameter. And area under the curve (AUC) was compared by *Z* test. The correlation between each parameter and proliferation index was calculated by Spearman correlation analysis. All statistical analyses were performed with SPSS (Version 19.0.0, IBM, Armonk, NY, United States) and MedCalc (Version 15.8, MedCalc Software, Acacialaan, Ostend, Belgium). *P* < 0.05 was considered to connote statistical significance.

## Results

### Patient Characteristics and Demographics

In this study, there were 42 patients with IDH1-mutant glioma and 49 patients with IDH1 wild-type glioma. Patients with IDH1-mutant glioma were younger than those with IDH1 wild-type glioma (*P* = 0.008). There was a significant difference of pathological grade distribution between IDH1-mutant group and IDH1 wild-type group (*P* < 0.001) (Table 1).

**TABLE 1:** Patient characteristics and demographics.

**Characteristics**	**IDH1-Mut**	**IDH1-WT**	***P*-value**
No. of patients	42	49	NA
Age (years), median (interquartile range)	53 (46.5, 58)	41 (34.75, 50.25)	0.001
Sex, *n* (%)			0.405
Male	20 (47.6%)	28 (57.1%)	
Female	22 (52.4%)	21 (42.9%)	
Pathological grading, *n* (%)			<0.001
II	19 (45.2%)	8 (16.3%)	
III	13 (31.0%)	7 (14.3%)	
IV	10 (23.8%)	34 (69.4%)	

*F, female; M, male; Mut, mutant; WT, wild-type; NA, not applicable.*

### Correlation of Diffusion Magnetic Resonance Imaging Parameters With Isocitrate Dehydrogenase 1 Genotype and Grade

**FIGURE 1:**
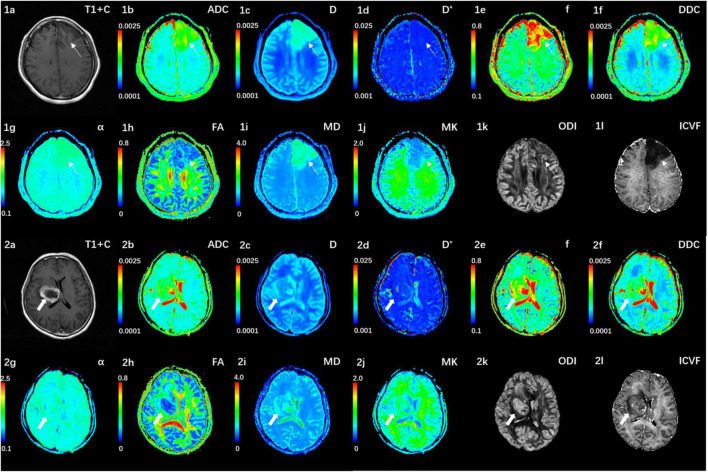
**(1a–l)** Correspond to a 38-year-old female with a IDH1-mutant grade III glioma in the left frontal lobe (white thin arrow). The ADC, MD, D, f, and DDC as well as α maps show increased values in the solid part of the tumor, while D*, MK, ODI, and ICVF maps show decreased values. **(2a–l)** Corresponds to a 59-year-old female with a IDH1 wild-type grade III glioma in the right basal ganglia (white thick arrow). The ADC, MD, D, f, and DDC as well as α maps show decreased values in the solid part of the tumor, while D*, MK, ODI, and ICVF maps show increased values. ADC, D, D*, and DDC as well as MD are in units of 10^–3^ mm^2^/s.

**FIGURE 2:**
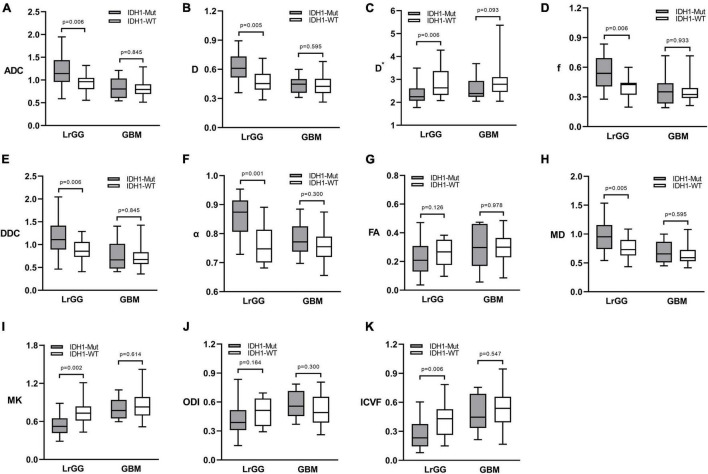
Box and whisker plots of DWI **(A)**, IVIM **(B–D)**, stretched-exponential DWI **(E,F)**, DTI **(G,H)**, DKI **(I)**, and NODDI **(J,K)** parameter in LrGGs and GBMs stratified according to IDH1 genotype. Boxes represent the median ± quartiles, with whiskers extending to the maximum and minimum values. ADC, D, D*, and DDC as well as MD are in units of 10^–3^ mm^2^/s. Mut, mutant; WT, wild-type; LrGG, lower-grade glioma; GBM, glioblastoma.

**FIGURE 3:**
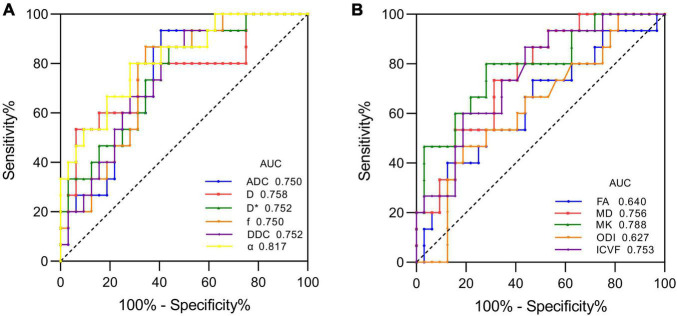
ROC curves of each parameter for distinguishing mutant and wild-type IDH1 in LrGGs. **(A)** ROC curves of DWI, IVIM, and stretched-exponential DWI parameters. **(B)** ROC curves of DTI, DKI, and NODDI parameters.

Figure 1 shows the contrast-enhanced T1-weighted images and dMRI parameter maps of typical IDH1-mutant and IDH1 wild-type glioma patients. In grade II and grade III lower-grade gliomas (LrGGs), IDH1 wild-type group showed significantly lower ADC, D, f, DDC, α, and MD values (*P* < 0.05) and higher D^∗^, MK, and ICVF values (*P* < 0.05) than IDH1-mutant group. However, there was no significant difference in FA and ODI values (*P* = 0.126 and 0.164, respectively) between the two groups (Figure 2). Table 2 shows the AUC values, sensitivity, specificity, and cutoff values for discriminating IDH1 genotype in LrGGs. ADC, D, D^∗^, f, DDC, α, MD, MK, and ICVF all showed high diagnostic efficacy in predicting IDH1 genotype in LrGGs; however, their AUC values were not significantly different (*P* > 0.05) (Figure 3). For glioblastomas (GBMs), no significant difference of these parameters was found between IDH1-mutant and IDH1 wild-type groups.

**TABLE 2:** ROC results of dMRI-derived parameter in differentiating IDH1 genotype in LrGGs.

**Parameters**	**AUC**	**Sensitivity %**	**Specificity %**	**Cut-off value**
ADC	0.750	59.38	93.33	1.084
D	0.758	68.75	80.00	0.553
D^*^	0.752	56.25	86.67	2.258
f	0.750	65.62	86.67	0.454
DDC	0.752	59.38	86.67	1.085
α	0.817	71.87	80.00	0.814
FA	0.640	87.50	40.00	0.318
MD	0.756	68.75	73.33	0.819
MK	0.788	71.87	80.00	0.613
ODI	0.627	81.25	46.67	0.518
ICVF	0.753	56.25	86.67	0.240

ADC, D, D*, DDC, and MD are in units of 10^–3^ mm^2^/s.

The dMRI-derived parameters of tumor lesions in LrGGs and GBMs in the presence of the same IDH1 genotype are presented in Table 3. In IDH1-mutant group, all parameters of dMRI, except D^∗^, and FA, can significantly distinguish LrGGs from GBMs (*P* < 0.05). In the IDH1 wild-type group, only ADC showed a critical statistical difference between LrGGs and GBMs (*P* = 0.048).

**TABLE 3:** dMRI-derived parameters in LrGGs and GBMs under the same IDH1 genotype.

**Parameters**	**IDH1-Mut**			**IDH1-WT**		
	**LrGGs**	**GBMs**	***P*-value**	**LrGGs**	**GBMs**	***P*-value**
ADC	1.142 (0.958, 1.436)	0.803 (0.606, 1.035)	0.003	0.963 (0.799,1.046)	0.792 (0.693,0.907)	0.095
D	0.611 (0.516, 0.732)	0.447 (0.358, 0.499)	<0.001	0.452 (0.390.0.553)	0.425 (0.354,0.503)	0.186
D^*^	2.242 (2.068, 2.611)	2.383 (2.241, 2.937)	0.140	2.630 (2.324,3.363)	2.791 (2.453,3.101)	0.558
f	0.539 (0.406, 0.693)	0.352 (0.234, 0.438)	0.007	0.425 (0.321,0.442)	0.325 (0.289,0.391)	0.165
DDC	1.110 (0.893,1.415)	0.665 (0.477, 1.017)	0.003	0.855 (0.730,1.060)	0.675 (0.575,0.834)	0.083
α	0.874 (0.807, 0.914)	0.771 (0.738, 0.825)	0.005	0.747 (0.701, 0.814)	0.755 (0.719, 0.790)	0.712
FA	0.208 (0.129, 0.307)	0.297 (0.169, 0.462)	0.125	0.267 (0.176, 0.352)	0.299 (0.229, 0.364)	0.386
MD	0.953 (0.740, 1.156)	0.655 (0.509, 0.865)	0.003	0.731 (0.629, 0.897)	0.590 (0.530, 0.724)	0.048
MK	0.522 (0.417, 0.649)	0.771 (0.647, 0.939)	<0.001	0.730 (0.613, 0.836)	0.827 (0.692, 0.984)	0.159
ODI	0.388 (0.309, 0.517)	0.556 (0.455, 0.714)	0.015	0.513 (0.351, 0.636)	0.491 (0.386, 0.655)	0.720
ICVF	0.233 (0.145, 0.376)	0.446 (0.334, 0.687)	0.002	0.430 (0.262, 0.528)	0.537 (0.392, 0.657)	0.073

Each parameter is presented as median (interquartile range). Mut, mutant; WT, wild-type; LrGGs, lower-grade gliomas; GBMs, glioblastomas. *means the parameter “fast ADC”.

### Correlation Between Diffusion Magnetic Resonance Imaging Parameters and Cell Proliferation Index

**FIGURE 4:**
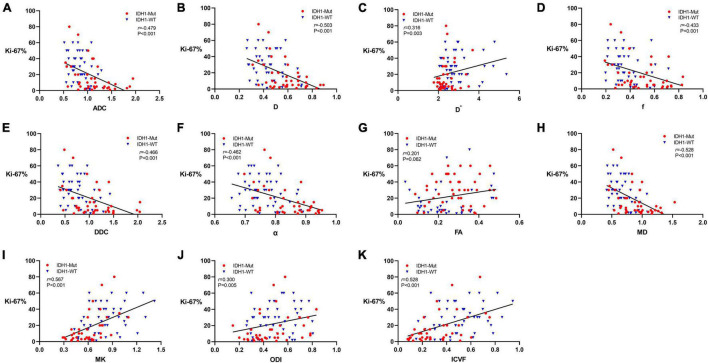
Scatter diagrams demonstrating the correlations between Ki-67 labeling index and parameters of DWI **(A)**, IVIM **(B–D)**, stretched-exponential DWI **(E,F)**, DTI **(G,H)**, DKI **(I)**, and NODDI **(J,K)**. And *r* represents the Spearman correlation coefficient.

The expression of Ki-67 LI was detected by immunohistochemical staining in 87 patients in this study. Figure 4 shows the Spearman correlation between Ki-67 LI and dMRI parameters in gliomas. Significant positive correlation was found between Ki-67 LI and D^∗^ (*r* = 0.316, *P* = 0.003), MK (*r* = 0.567, *P* < 0.001), ODI (*r* = 0.300, *P* = 0.005) as well as ICVF (*r* = 0.528, *P* < 0.001). In contrast, ADC, D, f, DDC, α and MD showed a significant negative correlation with Ki-67 LI (*r* = −0.479, *P* < 0.001; *r* = −0.503, *P* < 0.001; *r* = −0.433, *P* < 0.001; *r* = −0.466, *P* < 0.001; *r* = −0.462, *P* < 0.001; *r* = −0.528, *P* < 0.001, respectively). FA had no significant correlation with Ki-67 LI (*r* = 0.201, *P* = 0.062). MK has the maximal correlation coefficient and D^∗^ has the minimal.

## Discussion

Our study showed the six diffusion models all provide at least one parameter with effective prediction performance on the IDH1 genotype in LrGGs. Also, dMRI parameters are promising in the assessment of cell proliferation, especially maximal correlation coefficient, found between MK and proliferation index.

Radiological differences were found between IDH1-mutant and wild-type groups in LrGGs. We suspected that IDH1 wild type existed in the LrGGs when more complex organizational structure, more abundant microvasculature, higher cell density, and more diffusion barriers appeared. However, none of the parameters showed significant differences in the identification of IDH1-mutant and wild-type groups in GBMs due to a single parameter with insufficient ability to discriminating diffusion and perfusion patterns of highly malignant and structurally complex. This hints that we should further explore differences in the GBM groups using a combination of multiple parameters in the future.

Previous studies have demonstrated the ability of conventional DWI and DTI to distinguish IDH-mutant gliomas from IDH wild-type gliomas (Xiong et al., 2016; Wu et al., 2018). In our study, ADC and MD values of IDH1 wild-type group were significantly lower than that of IDH1-mutant group in LrGGs. FA showed ineffective prediction on IDH1 genotype, possibly due to the high heterogeneity in FA values for the solid component of the tumor, which is consistent with the results of Tan et al. (2019).

Many studies have found that DKI has excellent efficacy in the grading diagnosis, differential diagnosis, molecular marker prediction, and prognostic assessment of glioma (Jiang et al., 2015; Pang et al., 2016; Hempel et al., 2018, 2019; Tan et al., 2019; Zhang et al., 2019; Haopeng et al., 2020). A DKI parameter MK reflects the complexity of the tissue microenvironment under the assumption of non-Gaussian distribution in the organism. Similar to the results reported by Zhao et al. (2019), our finding showed MK effectively discriminated IDH1-mutant group from wild-type group in LrGGs. IDH1-mutant gliomas have lower cell density, lower tissue complexity, and less aggressiveness, so they have decreased MK values, while IDH1 wild-type gliomas have a more heterogeneous tissue microenvironment, higher cell density, and more aggressive phenotype, resulting in significantly increased MK values.

Orientation dispersion index and ICVF computed based on the NODDI model represent neurite dispersion characteristics and neurite density, respectively. Maximov et al. (2017) found that two index parameters, ICVF and ODI, could identify low-grade glioma and high-grade glioma, and ICVF had the highest diagnostic efficacy and was significantly better than DTI. Li et al. (2019) considered NODDI to be a promising method for grading gliomas and predicting cell proliferation. Diffuse overgrowth of tumor cells with increased density leads to an increase in ICVF values. In addition, tumor cells grow infiltratively along adjacent vessels and around nerve axons, often accompanied by degradation and destruction of white matter nerve fiber bundles, causing microstructural changes and elevated dispersion in axonal bundles, leading to increased ODI. Our results showed only ICVF significantly distinguished IDH1-mutant group from IDH1 wild-type group in LrGGs. IDH1 wild-type gliomas may be more proliferative and aggressive due to more complex microstructure and thus possess higher ICVF values. Zhao et al. (2018) reported that the mean ICVF was significantly higher in GBMs with IDH1 mutation than that without IDH1 mutation. However, only four cases of IDH1-mutant GBMs were collected in their study, and the findings still need to be confirmed as even different to ours.

The IVIM model was proposed by Le Bihan et al. (1986), where D represents the diffusion movement of water molecules inside and outside the cell, D^∗^ reflects the blood perfusion of the microcirculation, and f represents the abundance of capillaries in the tissue. In recent years, IVIM has been widely used in the grading and differential diagnosis of glioma, reflecting microscopic features such as tumor cell density and vascular proliferation (Bai et al., 2016; Shen et al., 2016; Kusunoki et al., 2020; Jiang and Minh Duc, 2021). Shen et al. (2016) studied the predictive efficacy of IVIM for glioma grading and considered that IVIM may be an imaging approach that combines arterial spin labeling (ASL) and ADC to assess tumor perfusion and diffusion. Minh Duc (Jiang and Minh Duc, 2021) found that IVIM has excellent diagnostic performance for distinguishing pilocytic astrocytoma from ependymoma. Increased D and f can reflect the pathological characteristics of pilocytic astrocytoma with low cell proliferation and high microvessel density, while decreased D and f reflects the high cell proliferation and low microvessel density of ependymoma. In the current study, the performance of D was slightly better than that of ADC in identifying the mutation status of IDH1 genotype in LrGGs perhaps due to D eliminates the influence of perfusion and more accurately reflects the diffusion and movement of water molecules. The D^∗^ value of IDH1 wild-type group is higher than that of IDH1-mutant group in LrGGs, indicating that IDH1 wild-type glioma has more abundant blood perfusion. Furthermore, *f*-value is higher in IDH1-mutant gliomas, inconsistent with Wang et al. (2019). The same contradictory results exist in studies of glioma grading, where *f*-values are higher in low-grade gliomas than in high-grade gliomas (Hu et al., 2014). Le Bihan (2019) suggested that the IVIM model is sensitive to fluid flow distributed within any voxel, not just blood flow. More relatively unrestricted water molecules outside the IDH1-mutant glioma cells may have contributed to the increase of *f*-values. Alternatively, these differences may be due to different IVIM model parameters, fitting methods, and ROI plotting methods (Cho et al., 2015).

The α is a parameter in stretched-exponential DWI that reflects the heterogeneity of the tissue. The high heterogeneity of tumor tissue, such as heterogeneous cells and proliferating vessels, leads to a decrease in α value (Chakhoyan et al., 2018; Chen et al., 2018). The DDC is a parameter reflecting diffusion in the stretched-exponential DWI, which is the weighted sum of ADC values. It can overcome the limitations of the biexponential DWI model regarding the assumption of fast and slow diffusion to reflect the diffusion characteristics of glioma, which is negatively correlated with tumor density. The stretched-exponential DWI model showed excellent efficacy in IDH1 genotype discrimination in our study, and α was able to distinguish IDH1 mutation status in LrGGs with the largest AUC value and high sensitivity and specificity. Lower α values indicate that the diffusion of water molecules in the tissue was inhomogeneous, and the heterogeneity of the tissue was higher (Bai et al., 2016). We speculate that the microenvironment of IDH1 wild-type glioma is more complicated, such as cell swelling and vascular proliferation, so it exhibits greater heterogeneity of intra-voxel diffusion.

In the ROC analysis of the diffusion parameters, we found that ADC, D, D^∗^, f, DDC, α, MD, MK, and ICVF were all characterized by higher specificity and lower sensitivity in identifying LrGG IDH1 genotypes. Of these, α and MK had the highest sensitivity and AUC values. We therefore considered that α and MK may be more appropriate diffusion parameters for identifying LrGG IDH1 genotypes.

This is of great clinical importance for the prediction of IDH1 wild-type LrGGs, which have a malignant clinical course despite being pathologically relatively inert alterations. Therefore, accurate and non-invasive prediction of the IDH1 genotype in LrGGs allows for timely treatment planning to impede malignant transformation of the disease.

We also investigated the prediction of glioma grading by dMRI under the same IDH1 genotype. Generally speaking, high-grade gliomas tend to be more heterogeneous, as shown in our findings. However, IDH1 wild-type LrGGs and GBMs showed only marginally statistically different ADC. Some studies have found that even in patients with IDH wild-type LrGG, tumors exhibit high levels of aggressiveness, with overall survival times similar to those of IDH wild-type GBM (Eckel-Passow et al., 2015; Wijnenga et al., 2017). This may explain our results, probably because the similar high heterogeneity and aggressiveness of IDH1 wild-type gliomas, resulting in most parameters that do not differ significantly between LrGGs and GBMs both with wild-type IDH1. In a word, with the increase of pathological grade, the tumor microstructure is more complex, with higher cell density and more disturbed water molecule movement, but IDH1 gene phenotype will affect the development of gliomas at a microscopic point of view. Compared with previous pathological grading studies (Bai et al., 2016), our study combined the pathological grading of gliomas with molecular phenotypes, which contributes to a more comprehensive understanding of the characteristics and microstructure of gliomas.

Nuclear protein Ki-67 is associated with cell proliferation specifically expressed in tumor cells (Alexiou et al., 2010; Gates et al., 2019). As the malignancy of the tumor increases, the blood supply becomes more abundant, the number of cells increases, malignant biological behavior ensues, such as hemorrhage and necrosis, and neovascularization forms further (Raab et al., 2010; Tietze et al., 2015). And these aforementioned alterations can affect the complexity and heterogeneity of tumor microstructure, when cellular gaps are smaller, water molecules diffusion is more restricted, and movement is more disturbed. Zhang et al. (2018) found MK and D have considerable potential to predict the degree of proliferation in diffuse astrocytomas. This is similar to our findings, where MK has maximal correlation coefficient with cell proliferation index. However, no significant correlation was found between FA and Ki-67 LI, which may be because the level of cell proliferation in response to Ki-67 only affects the size of the diffusion and not the pattern of diffusion routes.

We studied the predictive efficacy of multiple dMRI parameters for glioma IDH1 genotype and cell proliferation and found their great potential. However, dMRI still has some limitations that hinder its applicability in the routine clinical diagnosis of gliomas. There is currently no consensus on the optimal protocol parameters and post-processing methods for dMRI. This concerns acquisition parameters (e.g., number of directions, *b*-values) as well as post-processing methods. Further technical and methodological advances in the field of dMRI are necessary. Standardization and validation criteria for dMRI acquisition and post-processing techniques are needed in order to improve comparability between research centers.

Our study had some limitations. First, the sample size was small, especially for IDH1 wild-type LrGGs and IDH1-mutant GBMs. Although this is related to the uneven distribution of IDH1 mutation status in gliomas, future prospective studies with large samples are needed to ensure the accuracy of the experiment. Second, only one molecule, IDH1, was considered in this study, but many other molecules status such as 1p/19q codeletion and O6-methylguanine-DNA methyltransferase promoter methylation also play an important role in the development of gliomas, which needs to be further investigated. Finally, the ROI in this study was placed on the solid part of the tumor tissue; although this approach is more flexible and can better avoid areas of hemorrhage, necrosis, and calcification, it does not consider the tumor tissue as a whole. In the future, we can outline 3D ROIs on the parenchymal part of the whole tumor to validate our results of this study.

## Conclusion

Our findings reveal the relationship between dMRI parameters and IDH1 genotype and proliferation index in gliomas. dMRI has great potential to provide imaging markers that are sensitive to microstructural changes in gliomas caused by IDH1 mutation and cell proliferation, thereby facilitating prognostic prediction and treatment of glioma patients.

## Data Availability Statement

The raw data supporting the conclusions of this article will be made available by the authors, without undue reservation.

## Ethics Statement

The studies involving human participants were reviewed and approved by the Ethics Committee of Tongji Hospital, Tongji Medical College, Huazhong University of Science and Technology. Written informed consent to participate in this study was provided by the participants’ legal guardian/next of kin.

## Author Contributions

YX and WZ: guarantor of integrity of the entire study. YX, SL, and WZ: study concepts and design. YX and NS: literature research. YX and TG: experimental studies and data analysis. YX, SZ, WL, and SL: manuscript preparation and editing. All authors contributed to the article and approved the submitted version.

## Conflict of Interest

The authors declare that the research was conducted in the absence of any commercial or financial relationships that could be construed as a potential conflict of interest.

## Publisher’s Note

All claims expressed in this article are solely those of the authors and do not necessarily represent those of their affiliated organizations, or those of the publisher, the editors and the reviewers. Any product that may be evaluated in this article, or claim that may be made by its manufacturer, is not guaranteed or endorsed by the publisher.

## Footnotes

1
https://www.nitrc.org/projects/noddi_toolbox
